# Risk factors for recurrence after keloid surgery with electron radiotherapy

**DOI:** 10.1097/MD.0000000000035683

**Published:** 2023-10-27

**Authors:** Chunlei Liu

**Affiliations:** Chifeng Municipal Hospital, Chifeng Clinical Medical School of Inner Mongolia Medical University, Chifeng, China.

**Keywords:** electron radiotherapy, keloid, recurrence, triamcinolone

## Abstract

The aim of this study was to investigate the effect of postoperative electron radiotherapy (RT) on local control for keloids and to identify risk factors for recurrence. The clinical data of 82 patients treated at our institution from January 2015 to October 2019 were collected. The data included the general condition of the patients, clinical characteristics of the keloids, treatment plan, local control, and treatment side effects. A total of 82 patients (129 keloids) were included. The study included 23 men (28%) and 59 females (72%). The median patient age was 32 years (range, 18–67 years). Twenty-six recurrences were observed, and the 1-, 3-, and 5-year local control rates were 93%, 81%, and 73%, respectively. Univariate analysis revealed that age (*P* = .03), hypertension (*P* = .04), scar shape (*P* < .001), primary site (*P* = .02), maximum lesion diameter (*P* < .001), pain and itching (*P* = .005), local tension (*P* = .005), and infection (*P* < .001) were risk factors for local recurrence. Multivariable analysis revealed that maximum lesion diameter (*P* < .001), infection (*P* < .001), interval between surgery and RT (*P* = .02), and previous treatment (*P* = .02) were independent risk factors. Complete excision of keloids combined with electron RT is safe and seemingly effective. For keloids with a high risk of recurrence, more aggressive treatment should be chosen, and further prospective studies are needed to explore the optimal treatment.

## 1. Introduction

A keloid is a pathological lesion caused by abnormal tissue repair due to excessive collagen deposition beyond the edge of the original injury. Keloid formation is believed to be caused by unbalanced repair during the wound healing.^[1,2]^ Surgical resection alone can reportedly lead to a local recurrence rate of more than 50%,^[2]^ and many combinations of methods have been used for the clinical treatment of keloids.^[3]^ Postoperative adjuvant radiotherapy (RT) is considered one of the most effective comprehensive treatments; it has a better curative effect than surgery or RT alone and is usually used in the clinical treatment of refractory and recurrent keloids secondary to other treatments (such as corticosteroids and local injections, laser treatment, or cryotherapy).^[1,4,5]^ Previous studies have shown that recurrence factors associated with keloid treatment include maximum diameter, primary site of hypertonia, previous treatments, family history, burn history, and complications of scar infection.^[6,7]^ However, whether age, sex, interval between surgery and RT, treatment after surgical adjuvant RT, local symptoms, and history of hypertension affect the efficacy of keloid treatment remains controversial. Therefore, the purpose of this study was to explore the local control of keloids via postoperative adjuvant external electron irradiation, discover risk factors for keloid recurrence, evaluate the effectiveness of adding triamcinolone acetonide as an adjuvant therapy to prevent keloid recurrence, and provide supporting data for individualized clinical treatment of keloids.

## 2. Methods

### 2.1. Patient characteristics

From January 2015 to October 2019, 82 patients (23 men and 59 women) with 129 keloids were treated at our institution. We retrospectively reviewed their clinical data. The median age of the patients undergoing treatment was 32 years (range, 18–67 years). All keloids were completely extirpated and histopathologically diagnosed prior to RT. Details of the patients and keloids are shown in Tables 1 and 2, respectively. The study was approved by the ethics committee of Chifeng Municipal Hospital.

**Table 1:** Characteristics of the study population.

Characteristic	No. (%)
Gender	
Male	23 (28%)
Female	59 (72%)
Median age (range) (yr)	32 (18–67)
Hypertension	
With	20 (24.4%)
Without	62 (75.6%)
Median lesion diameter (range) (cm)	3.6 (0.5–21)
Number of keloids	
Single	46 (56.1%)
Multiple	36 (43.9%)

**Table 2:** Prognostic characteristics of keloids.

Characteristic	No. (%)
Keloids	129
Primary location	
Earlobe	55 (42.6%)
Other location	74 (57.4%)
Shape of lesions	
Regular	74 (57.4%)
Irregular	55 (42.6%)
Infection	
With	34 (26.4%)
Without	95 (73.6%)
Local tension	
Low	95 (73.6%)
High	34 (26.4%)
Diameter	
≤4 cm	82 (63.6%)
>4 cm	47 (36.4%)
Pain and itching	
With	79 (61.2%)
Without	50 (38.8%)
Previous treatment	
Yes	78 (60.5%)
No	51 (39.5%)
Interval between surgery and RT	
≤24 h	63 (48.8%)
>24 h	66 (51.2%)
BED (Gy) (α/β = 10)	
19.5	58 (45.0%)
22.5	38 (29.5%)
28	33 (25.5%)
Adjuvant triamcinolone acetonide	
Yes	45 (34.9%)
No	84 (65.1%)

RT = radiotherapy. BED = biologically effective dose.

### 2.2. Surgical treatment

To receive adjuvant RT after surgical resection at our institution, patients must make an appointment in advance. To accurately determine the target area for RT, surgeons work together with radiation oncologists to mark the keloid locations.

Hemostasis was achieved using electrocautery, and the wounds were closed without tension. All excised specimens were subjected to independent histological analyses to determine the diagnosis. The patient was transferred to the Department of Radiation Oncology for development of a RT plan and receiving the initial treatment.

### 2.3. Radiotherapy

Radiotherapy was initiated as soon as possible for all patients after comprehensive evaluation. For earlobe lesions, the first RT session was performed within 2 hours of the surgery. For other lesions, the surgeon and RT evaluated the wound and decided when to start the first radiation. Radiotherapy was performed using a 6-MeV electron beam generated by a linear accelerator (Varian 21EX) with full shielding to protect normal tissues. Before treatment, RT oncologists applied 0.5 cm of wax to increase the surface dose. The target extended to 0.5 to 0.8 cm outside the surgical incision, including 0.3 to 0.5 cm outside the needle hole of the surgical suture, and if necessary, the boundary was extended to 1.0 cm (if the surgeon thought that the area contained a residual lesion). The prescribed doses for these sections were 5 Gy × 3 fractions, 3 Gy × 5 fractions, and 4 Gy × 5 fractions.

### 2.4. Follow-up and statistical analysis

Patients were required to regularly return to the hospital for face-to-face follow-ups. Patients who could not return for in-hospital follow-up were contacted by phone to confirm the success of the keloid treatment. Follow-up visits were scheduled at 3 months, 6 months, 1 year, and every year thereafter. All patients were followed up for at least 1 year after treatment to improve the validity of the outcome, because the chance of recurrence is minimal after 1 year.^[8,9]^ Statistical analysis was performed with the IBM SPSS Statistics for Windows version 19.0 (IBM Corp.). The Kaplan–Meier method and multivariable Cox regression analysis were used to compare the local control rate and evaluate risk factors for relapse, respectively.

## 3. Results

**Figure 1.**
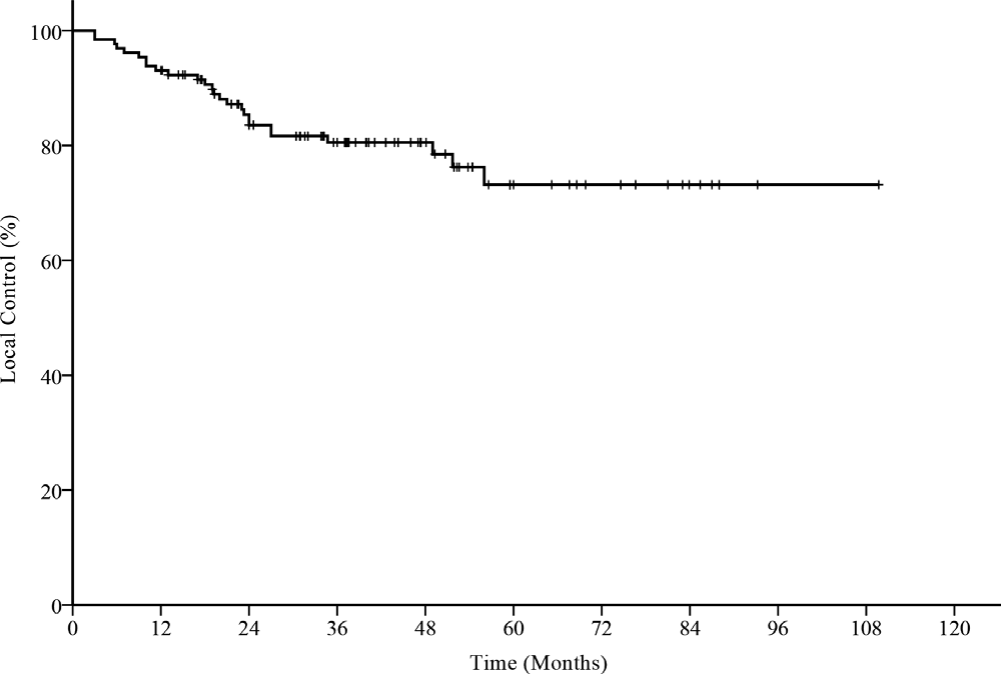
Overall local control of 129 keloids.

**Figure 2.**
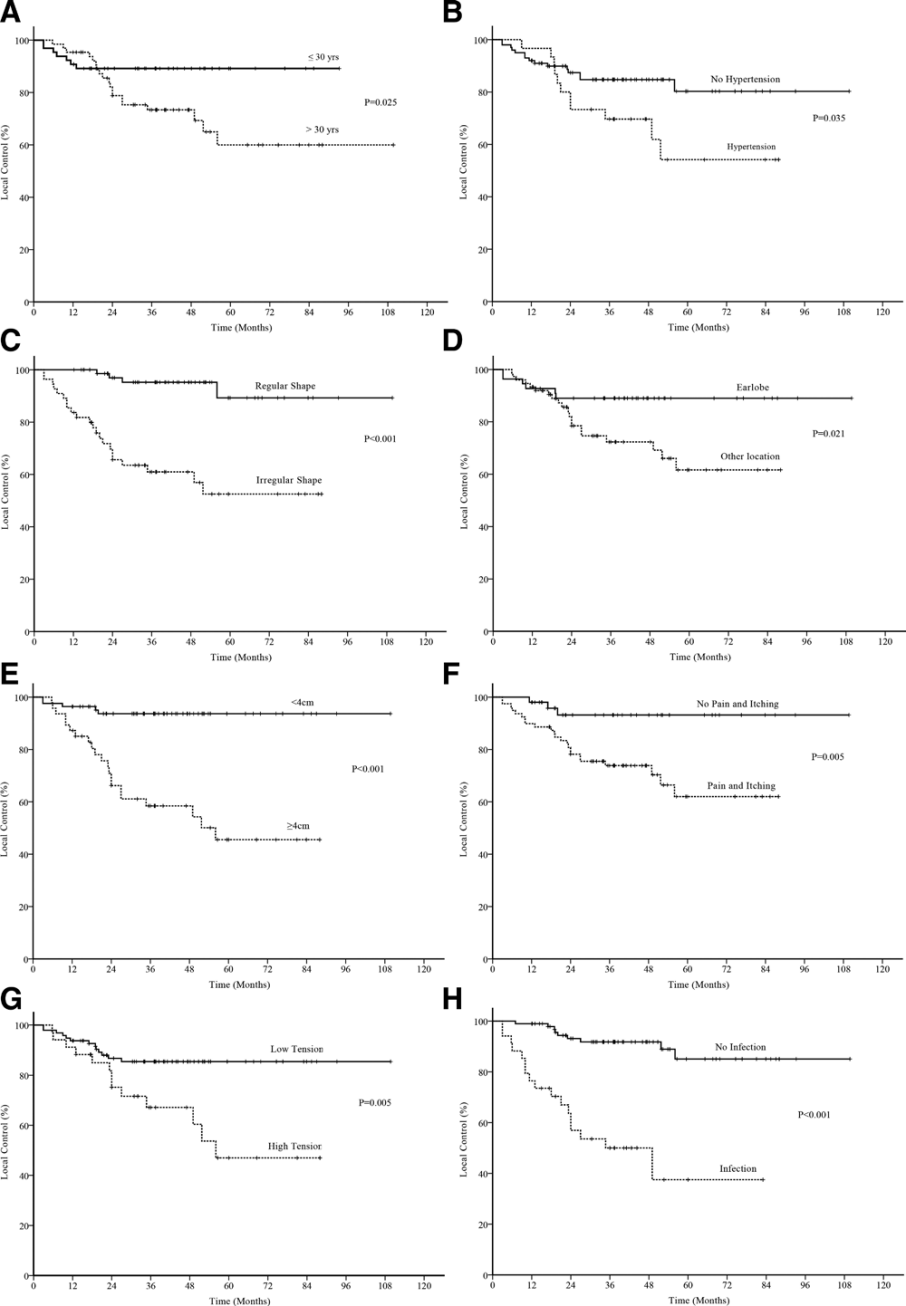
Factors affecting local control of postoperative electron radiotherapy for keloids: (a) age, (b) hypertension, (c) shape of lesions, (d) primary site, (e) maximum diameter of the lesion, (f) pain and itching symptoms, (g) local tension, and (h) infection.

Several prognostic factors were analyzed, as demonstrated in Table 2. Twenty-six recurrences were observed, and the 1-, 3-, and 5-year local control rates were 93%, 81%, and 73%, respectively (Fig. 1). Univariate analysis revealed that age (*P* = .03), hypertension (*P* = .04), scar shape (*P* < .001), primary site (*P* = .02), maximum lesion diameter (*P* < .001), pain and itching (*P* = .005), local tension (*P* = .005), and infection (*P* < .001) were risk factors for local recurrence (Fig. 2). Other factors, including sex, number of keloids, erythema or acne, previous treatment, adjuvant triamcinolone acetonide treatment, and interval between surgery and RT, exhibited no effect on local control (Table 3). In the multivariable logistic regression analysis, the maximum diameter (*P* < .001), infection (*P* < .001), interval between surgery and RT (*P* = .02), and previous treatment (*P* = .02) were significantly associated with local control (Table 4).

**Table 3:** Factors that have no impact on local control.

Characteristic	Local control rate			*P* value
	1 year (%)	3 year (%)	5 year (%)	
Gender				
Male	85	74	74	.408
Female	84	83	73	
Number of keloids				
Single	96	79	73	.982
Multiple	91	82	74	
Empyrosis or acne				
Yes	92	72	59	.117
No	94	83	79	
Previous treatment				
Yes	91	87	80	.079
No	94	72	60	
Adjuvant triamcinolone acetonide				
Yes	94	79	73	.978
No	93	81	73	
Interval between surgery and RT				
≤24 h	92	87	82	.124
>24 h	94	73	64	

RT = radiotherapy.

**Table 4:** Logistic multivariate analysis of keloid recurrence.

Variables	HR	95% CI		*P* value
Diameter	0.085	0.025	0.291	.000
Infection	8.001	3.110	20.582	.000
Interval between surgery and RT	3.667	1.193	11.269	.023
Previous treatment	0.377	0.165	0.857	.020

CI = confidence interval, HR = hazard ratio, RT = radiotherapy.

After treatment, 4.7% (6/129) of the keloids (2 in the anterior chest, 2 in the shoulder, and 1 in the abdomen) exhibited acute RT-related side effects, mainly manifesting as delayed wound healing, but all healed completely within 1 month of RT. A keloid located on the back developed local infection, which resolved after anti-infective treatment and did not relapse. One patient with a keloid on the earlobe developed grade I dry peeling and a typical hypertrophic scar after RT. The hypertrophic scar gradually subsided over 2 years, and no local recurrence was observed at the last follow-up. No late complications occurred in any of the patients.

## 4. Discussion

A keloid is a type of benign neoplastic disease in the form of a pathological scar. Its pathogenesis is mainly due to an uncontrolled increase in collagen synthesis. It causes local symptoms, such as pruritus, and impairs the patient’s quality of life. The main goals of treatment are to eliminate local symptoms, improve the appearance of the keloid, and prevent recurrence. Many traditional treatment options are available, and many novel targets and therapies have recently been explored.^[10]^ For small lesions, the preferred approaches include intralesional corticosteroids, silicone elastomer sheeting, cryotherapy, and pressure dressing.^[11,12]^ However, for resistant or recurrent lesions, extralesional surgical excision in combination with other treatments, especially adjuvant postoperative RT, is considered the most efficacious treatment, reducing the relapse rate to below 10% and improving patient quality of life.^[9,13,14]^ In our study, the 5-year local control rate was 73%, which is comparable to that of previous reports.

Despite reports of risk factors for keloid recurrence, the identification of patients at high risk of recurrence after postoperative RT remains a challenge for clinicians.^[15,16]^ We retrospectively analyzed the clinical data of patients with keloids who underwent postoperative electron RT. Multivariable analysis revealed that local infection, maximum scar diameter, previous treatment, and the interval between surgery and RT were independent risk factors for local recurrence. However, a history of hypertension, age, lesion shape, primary site, local tension, and pain and pruritus were not independent risk factors. In 1994, Klumpar et al^[17]^ reported the prognostic factors following postoperative RT for keloids. They followed patients for a median of 12 years, sufficiently long to evaluate recurrence. Their univariate analyses revealed that age <25 years was associated with a higher rate of recurrence after treatment. In contrast, in our study, the relapse rates were higher in patients older than 32 years. Moreover, Klumpar et al^[17]^ reported that patients with a family history of keloids and multiple lesions were more likely to relapse after treatment. However, in our study, only 3 patients had a family history of keloids, and none of them have exhibited signs of recurrence to date. A recent study of 75 keloid lesions treated with surgical excision and electron RT revealed that patients with multiple lesions had a worse local control rate than those with single lesions.^[18]^ However, in the present study, 36 patients had multiple keloids, and multiple lesions were not significant risk factors for local recurrence either in univariate or multivariable analyses. Prospective studies are required to confirm these results.

In this retrospective study, a large proportion of keloids occurred on the ears. All of the patients with ear keloids exhibited perfect local control, but multivariable analysis revealed that such a location was not an independent prognostic factor. A detailed review of the treatment history revealed that patients with ear keloids were treated more often with surgery alone; thus, surgeons should explore adjuvant strategies for patients with multiple relapses. Keloids in other locations were more irregular in shape, had high tension, and had a relatively low local control rate, which was similar to the results of previous studies.^[19–24]^

As in the general population, hypertension was more common among older patients in this study. Although univariate analysis suggested that hypertension was a prognostic factor for the need for postoperative adjuvant electron RT for keloids, multivariable analysis revealed that it was not an independent factor. In previous studies, hypertension also did not influence local control of keloids after comprehensive treatment.^[18,25]^ Large, prospective studies including more older patients with keloids are needed to confirm the lack of relationship between hypertension and keloid recurrence.

A previous study of 75 patients with 113 keloids revealed that keloids >2 cm and male sex yielded a higher rate of local recurrence.^[26]^ Because of the differences in grouping between our study and theirs, we discovered a lower local control rate for keloids >4 cm. Histopathological studies have revealed that keloid tissue exhibits increased infiltration of immune cells, especially macrophages and T lymphocytes.^[27,28]^ Other studies have revealed an important relationship between hypertension and the aggravation of pathological scars, possibly because the pressure on the newly formed blood vessels in the scars promotes the relaxation of local blood vessels, which in turn aggravates the local chronic inflammatory response.^[29,30]^ Therefore, the presence of inflammation is considered a risk factor for keloid formation, and an infection before treatment is considered a risk factor for recurrence after keloid treatment; however, the specific mechanism is not fully understood.

In this study, the local recurrence rate of keloids did not significantly differ between men and women, which differs from the results in a previous report.^[7]^ This may be owing to the large differences in the proportions of men and women enrolled in the different studies. A more balanced sex ratio is needed to analyze the relationship of sex with local control after comprehensive keloid treatment.

Based on the location of keloids, the RT segmentation scheme differed in our study. Ogawa et al reported on an Asian population in which 270 keloids and hypertrophic scars were treated with surgical resection combined with adjuvant RT.^[31]^ They concluded that RT should be tailored according to the primary keloid site. They recommended that 20 Gy should be applied in 4 fractions for lesions in the anterior chest, suprapubic region, and scapular region, and 10 Gy in 2 fractions for lesions in the earlobe. Other lesions can be treated with 15 Gy divided into 3 fractions.^[31,32]^ Our results were similar to those of the largest retrospective study, in which surgical removal of keloids was followed by 6- or 7-MeV external electron irradiation as adjuvant RT. The prescribed dose of RT was 18 Gy in 2 fractions, and the interval between the 2 fractions was 1 week. Postoperative RT is typically initiated between 24 and 48 hours after surgery for 10 to 15 days to ensure survival of the suppressed flap. An overall local control rate of 88.3% was achieved after a median follow-up period of 40 months in a previous study.^[6]^ As electron beam irradiation has satisfactory dose distribution and safety, it is one of the most widely used RT methods in clinical practice for keloids.

However, our study had several limitations, such as the small sample and the imbalance of certain factors. Therefore, more appropriate RT methods and segmentation schemes should be explored in the future to further improve therapeutic effects.

In conclusion, complete surgical resection combined with electron RT is a safe and seemingly effective treatment for keloids. The overall 5-year local control rate in our study was 73%. This study provides clinicians with useful information to make treatment decisions such as providing more aggressive treatment to patients at high risk of recurrence.

## Author contributions

**Formal analysis:** Chunlei Liu.

**Project administration:** Chunlei Liu.

**Writing – review & editing:** Chunlei Liu.
